# The impact of consumer demands and trends on food processing.

**DOI:** 10.3201/eid0304.970408

**Published:** 1997

**Authors:** D L Zink

**Affiliations:** Nestlé USA, Inc., Glendale, California 91203, USA. donald.zink@us.nestle.com

## Abstract

In the United States, consumer demand for new foods and changes in eating habits and food safety risks are affecting the food processing industry. The population is becoming older on average; moreover, consumers want fresh and minimally processed food without synthetic chemical preservatives. To address the need for safer food and compete for consumer acceptance, manufacturers are exploring new food processing and preservation methods.


### The Impact of Consumer Demands and Trends on Food Processing

**Don L. Zink**
Nestlé USA, Inc., Glendale, California, USA

In the United States, consumer demand for new foods and changes in eating habits and food safety risks are affecting the food processing industry. The population is becoming older on average; moreover, consumers want fresh and minimally processed foods without synthetic chemical preservatives. To address the need for safer food and compete for consumer acceptance, manufacturers are exploring new food processing and preservation methods.

### Fresh, Preservative-Free Foods That Promote Health

Food industry marketers perceive that consumers want foods that are convenient; fresh (less-processed and less-packaged); all natural—with no preservatives (a so-called “clean label”); without a perceived negative (i.e., foods without high fat, high salt, and high sugar); and healthy. The industry perception is that consumers want foods that not only cause no harm but also remedy ailments from heart disease, osteoporosis, and fatigue to memory loss. Categories of foods that promote health are fortified foods, performance-enhancing food additives, probiotics, and prebiotics.

Food fortification is an old process. Milk (with vitamins A and D), bread (with iron and niacin), or salt (with iodine) have long been fortified to replace nutrients thought to be lost during processing. Newer foods fortified with nutrients needed by the body to stave off the progression of diseases associated with aging or enhance physical performance attract the consumer’s attention and sell well in today’s marketplace. For example, marketers are promoting all sorts of foods fortified with calcium to women concerned about osteoporosis. Performance-enhancing foods are popular. Such foods range from beverages to replace electrolytes and prolong physical endurance to amino acids and fatty acids to improve alertness and memory. Probiotics and prebiotics are two paths to the same result. Research studies suggest that a desirable intestinal microflora causes the host to be less susceptible to intestinal pathogens. Probiotics create this desirable state by incorporating the microorganism directly into the food, either as a stable culture or as part of food fermentation. This process is costly, and the microorganisms often do not survive well in the food. Thus, manufacturers must add 10 to 100 times the needed number of microorganisms to account for a loss of viability during the product’s normal shelf life. Prebiotics overcomes the limitations of probiotics by adding specific nutrients, usually a particular carbohydrate, to the food. When ingested as part of the diet, these specific nutrients “select” for a beneficial microflora in the intestinal tract.

Address for correspondence: Don L. Zink, Nestlé USA, Inc. 800 N. Brand Blvd., Glendale, CA 91203, USA; fax: 818-549-6908; e-mail: donald.zink@us.nestle.com.

### Food Processing and Food Product Development

The consumer’s quest for health is having a great impact on the food processor. Compared with the marketplace of 25 years ago, today’s marketplace has more perishable products, including fruits and vegetables, and more innovative packaging. In addition, consumer aversion to traditional chemical preservatives has left food processors with less flexibility in choosing preservation methods. To find a technologic edge in the marketplace, food processors are exploring new processing and preservation technologies. Some of these technologies include ohmic heating, high-pressure, pulsed electric field, bright light, and aseptic processing. Ohmic heating involves passing an electric current through the food to create heat due to electrical resistance within the food. With ohmic heating, food particles heat at the same rate as the carrier medium or sauce. Ohmic heating can enhance food quality by limiting heat damage to the sauce and food particles. High-pressure processing uses very high pressure, often thousands of atmospheres, to pasteurize foods without heat. This technology is ideal for heat-sensitive foods, but some enzymes are difficult to inactivate with high-pressure processing. Pulsed electric field processing uses a very strong pulsed electric current to disrupt microbial cells and pasteurize foods with little or no heating. Bright light processing uses an intense white light to kill bacteria on the surface of foods; this light does not penetrate deeply into foods and can only be used for surface pasteurization.

Aseptic processing dates back to at least the mid-1940s but has yet to realize its full potential. The most widely used of these new technologies, aseptic processing involves sterilizing a food product in a continuous process through a heat exchanger and then filling that food in an aseptic filler. The aseptic filler is a highly specialized piece of equipment designed to sterilize the packaging material, fill the sterile product into its container in a sterile environment, and then seal the package.

Food processors have also explored novel food preservation systems. An ideal food preservative would come from a natural source and preserve food without being labeled a synthetic chemical preservative. Such preservatives include bacteriocins, dimethyl dicarbonate (Velcorin), competitive microbial inhibition, controlled and modified atmospheres, and irradiation. Bacteriocins are not new; however, like nisin, they are now being used to extend shelf life and enhance the safety of a variety of food products. The use of bacteriocins is likely to be expanded in the future. Dimethyl dicarbonate, a relatively new preservative used in beverages such as wine, tea, and juices, is particularly effective in preventing spoilage caused by yeasts. Competitive microbial inhibition relies on the fact that many harmless bacteria, notably lactic acid bacteria, can inhibit the growth of both spoilage bacteria and pathogens. Inhibitory strains of lactic acid bacteria can be selected for use in dairy cultures or be added to refrigerated foods to extend shelf life and enhance safety. Modified and controlled atmosphere packaging are already widely used by the food industry. They have the potential for even wider use, particularly with fresh fruits and vegetables sold at retail. These methods rely on inhibiting microbial growth by excluding oxygen or by inhibitory concentrations of carbon dioxide. Carefully selected gas mixtures can also delay the ripening of certain fruits and vegetables and extend the shelf life of fresh meats. Finally, irradiation, also not a new technology, is poised for widespread use to enhance the safety and shelf life of many foods. With proper controls, irradiation could be a valuable means of reducing Salmonella contamination of poultry and *Escherichia coli* O157:H7 contamination of ground beef.

### A Scientist's View of Consumer Trends

One of the most obvious consumer trends is a dramatic increase in the consumption of fresh foods, particularly fruits and vegetables. This increase is the result of the well-publicized value of a high-fiber diet and betacarotenes as an aid in preventing colon cancer. The number of meals eaten away from home has increased dramatically. The trend toward dining outside the home is likely rooted in lifestyle changes such as households with two working parents. The number of home-delivered meals, the ultimate convenience food, has also increased, even though the most popular foods consumed today (pizza and hamburgers) are generally the same as those of 20 years ago. This indicates that the types of foods consumed do not change rapidly, but the way these foods are consumed has changed. Finally, the population is getting older on average. Aging may not be a consumer trend, but it has a profound effect on food safety considerations. An older population means a more susceptible population.

New food processing and preservation technologies and wider applications of older technologies have, for the most part, had little impact on most processed foods. Adoption of new technologies will likely continue at a slow pace. Consumers consistently buy foods on the basis of value and taste, not processing technology. Technologies that add value will be the first to gain consumer acceptance. The demand for convenience foods will probably increase. Demands on our time are increasing, and we have less time to spend on food preparation, and more meals will be eaten away from home, in part because of convenience but also because of a trend for new tastes and variety in the diet.

Finally, the trend toward foods that claim to enhance performance, rooted in an aging population's need for better health during longer life-spans, will continue. With increased demand, the pressure on the food industry for better processing and preservation methods will also increase and may result in safer food.

